# Predictors of outcome after 6 and 12 months following anthroposophic therapy for adult outpatients with chronic disease: a secondary analysis from a prospective observational study

**DOI:** 10.1186/1756-0500-3-218

**Published:** 2010-08-03

**Authors:** Harald J Hamre, Claudia M Witt, Gunver S Kienle, Anja Glockmann, Stefan N Willich, Helmut Kiene

**Affiliations:** 1 Institute for Applied Epistemology and Medical Methodology, Zechenweg 6, 79111 Freiburg, Germany; 2 Institute of Social Medicine, Epidemiology, and Health Economics, Charité University Medical Center, 10098 Berlin, Germany

## Abstract

**Background:**

Anthroposophic medicine is a physician-provided complementary therapy system involving counselling, artistic and physical therapies, and special medications. The purpose of this analysis was to identify predictors of symptom improvement in patients receiving anthroposophic treatment for chronic diseases.

**Methods:**

913 adult outpatients from Germany participated in a prospective cohort study. Patients were starting anthroposophic treatment for mental (30.4% of patients, n = 278/913), musculoskeletal (20.2%), neurological (7.6%), genitourinary (7.4%) or respiratory disorders (7.2%) or other chronic indications. Stepwise multiple linear regression analysis was performed with the improvement of Symptom Score (patients' assessment, 0: not present, 10: worst possible) after 6 and 12 months as dependent variables. 61 independent variables pertaining to socio-demographics, life style, disease status, co-morbidity, health status (SF-36), depression, and therapy factors were analysed.

**Results:**

Compared to baseline, Symptom Score improved by average 2.53 points (95% confidence interval 2.39-2.68, p < 0.001) after six months and by 2.49 points (2.32-2.65, p < 0.001) after 12 months. The strongest predictor for improvement after six months was baseline Symptom Score, which alone accounted for 25% of the variance (total model 32%). Improvement after six months was also positively predicted by better physical function, better general health, shorter disease duration, higher education level, a diagnosis of respiratory disorders, and by a higher therapy goal documented by the physician at baseline; and negatively predicted by the number of physiotherapy sessions in the pre-study year and by a diagnosis of genitourinary disorders. Seven of these nine variables (not the two diagnoses) also predicted improvement after 12 months. When repeating the 0-6 month analysis on two random subsamples of the original sample, three variables (baseline Symptom Score, physical function, general health) remained significant predictors in both analyses, and three further variables (education level, respiratory disorders, therapy goal) were significant in one analysis.

**Conclusion:**

In adult outpatients receiving anthroposophic treatment for chronic diseases, symptom improvement after 6 and 12 months was predicted by baseline symptoms, health status, disease duration, education, and therapy goal. Other variables were not associated with the outcome. This secondary predictor analysis of data from a pre-post study does not allow for causal conclusions; the results are hypothesis generating and need verification in subsequent studies.

## Background

Chronic diseases are the most common cause of disease burden worldwide and are rarely completely cured [1]. Strategies to improve the outcome of chronic diseases include new drug regimens, enhanced healthcare provision, and patient self-management programs [2-4]. Many patients with chronic disease also use complementary therapies [5,6]. It is important to know which types of patients will use complementary therapies and which patients will profit from such use.

Anthroposophic medicine (AM), founded by Rudolf Steiner and Ita Wegman [7], is a physician-provided complementary therapy system. AM acknowledges a spiritual-existential dimension in man, which is assumed to interact with psychological and somatic levels in health and disease. AM therapy for chronic disease aims to counteract constitutional vulnerability, stimulate salutogenetic self-healing capacities, and strengthen patient autonomy [8-10]. This is sought to be achieved by counselling [9]; by non-verbal artistic therapies using painting or clay [11,12], music [13] or speech exercises [14]; by eurythmy movement exercises [15]; by physical therapies [16,17]; and by special AM medications. Worldwide, AM physicians work in 56 countries [18].

Patients using AM for chronic disease are predominantly women or children, education and occupation levels are higher than average, and typical indications are mental, respiratory, and musculoskeletal disorders [9,10,19-21]. Studies of AM therapy for different cancers [22-24] and facial neuralgia [25] have identified outcome differences according to gender [23,25], psychosomatic self-regulation [22], and performance status [24].

The Anthroposophic Medicine Outcomes Study (AMOS) [20] provided an opportunity to assess a broad range of outcome predictors in AM therapy users. AMOS was a prospective, long-term cohort study of patients starting AM therapies for various chronic diseases. Following AM therapies, disease symptoms were reduced and quality of life improved, without cost increase [20,26]. Outcome differences have been assessed in univariate analyses [27-39]. We present here a multivariate analysis of predictors of symptom improvement in adult patients of the AMOS study.

## Methods

### Design and objective

This was a secondary analysis of data from a prospective observational cohort study conducted in German outpatient settings. The study was initiated by a health insurance company as part of a research program on the effectiveness, costs, and safety of AM therapies in chronic disease [20,26,40]. The purpose of the present analysis was to identify variables predicting clinical improvement following AM treatment for adults. For this purpose we performed multiple linear regression analyses, with the improvement of disease symptoms at 6- and 12-month follow-up as dependent variables, and with various items concerning disease status, socio-demographics, life-style, and therapies as independent variables.

### Setting, participants, and therapy

All physicians certified by the Physicians' Association for Anthroposophical Medicine in Germany and working in an office-based practice or outpatient clinic in Germany were invited to participate in the AMOS study. Certification as an AM physician required a completed medical degree and a three-year structured postgraduate training. The participating physicians recruited consecutive patients starting AM therapy. Patients enrolled in the period from 1 January 1999 to 31 December 2005 were included in the present analysis if they fulfilled the following criteria:

1. Outpatients aged 17-75 years.

2. Starting AM therapy for any indication (main diagnosis). 

2A: AM-related consultation of at least 30 minutes followed by new prescription of AM medication or other AM treatment administered by the physician. 

2B: or referral to AM treatment (art therapy, eurythmy therapy or rhythmical massage therapy).

3. Duration of main diagnosis of at least 30 days at study enrolment.

4. Symptom Score evaluable at baseline and at six-month-follow-up.

Symptom Score was a compound measure of the symptoms for which the patients had sought medical attention. At baseline, the patients documented one to six symptoms in order of decreasing importance and assessed the intensity of each symptom on a numerical rating scale from 0 ("not present") to 10 ("worst possible") [41]. At each follow-up, the patients documented the intensity of the same symptoms which they had documented at baseline. Symptom Score was the average severity of all documented symptoms per patient at each documentation point. This score has not been validated.

Patients were excluded if they had previously received the AM therapy in question (see inclusion criterion 2) for their main diagnosis. For analysis of patients according to AM therapy modality, patients fulfilling inclusion criteria 2a as well as 2b were allocated to group 2b.

### Variables

#### Dependent variable

Symptom Score was selected as dependent variable because it directly described the severity of the symptoms for which the patients had sought help, and because it was documented by the patients themselves. Symptom Score data were available after 3, 6, 12, 18 and 24 months; the change from baseline to 6-month follow-up was chosen for the main analysis because previous analyses [20,34-37] had shown that the AM therapies were mainly administered in the first 3-6 months, because most of the Symptom Score improvement occurred during the first 6 months, and since follow-up rates decreased progressively beyond 6 months.

#### Independent variables

Independent variables pertaining to socio-demographics, life-style, disease status at baseline, and therapy factors were selected from the dataset. Two types of variables were selected. The first type were factors for which a positive association with the outcome (e. g. better outcome among patients with higher education) was a-priori deemed to be possible:

• more favourable socio-demographic characteristics and healthier life-style (e. g. higher education, not smoking)

• more favourable health status at baseline (e. g. low degree of comorbidity, short disease duration, low depression scores)

• more experienced AM therapy providers (years since qualifications of AM physicians and therapists, respectively)

• previous treatment by the AM physician

• more intensive AM therapy (longer duration of first consultation with AM physician, more AM therapy sessions, more AM medications)

• more intensive non-AM therapy (e. g. more physiotherapy sessions, more non-AM medications etc)

For the second type of variables there was no reason to assume a priori a particular direction of an association (e. g. no reason to assume that women have better outcomes than men or vice versa):

• demographics (gender, age)

• baseline status (main diagnosis)

• treatment (primary care or other setting, main AM therapy modality)

Since a large data set was available, and since the literature and clinical reasoning did not suggest otherwise, the approach was deliberately broad, including as dependent variables all variables of interest according to the above criteria.

The original list of dependent variables had 47 items, all of which were used in the final analyses. During revision of the analyses, the issue of selection bias was more thoroughly investigated, prompting us to include one further dependent variable: physician's therapy goal for the patient at baseline. Thus a total of 48 items were included (corresponding to 61 variables including dummy variables, see Tables 1 and 2). Of these 48 items, 10 items (age, gender, diagnosis, disease duration, depression, number of patients enrolled per physician, main AM therapy modality, and the use of non-AM medications, psychotherapy and physiotherapy, respectively) had already been subject to bivariate analyses, most of which had failed to show relevant associations with clinical outcomes [28-39] (exceptions: different results of AM medication therapy in diagnostic subgroups [38] and different results of asthma therapy in adults vs. children [30]). The remaining 38 items were selected without prior knowledge about bivariate associations with outcome.

**Table 1:** Continuous and rank-ordered independent variables

Variable	N evaluable	Mean	Standard deviation	Correlation	
				
				R	P-value
Age (years)	913	44.38	12.07	-0.08	0.020

Education level (1 = low, 2 = intermediate, 3 = high)	913	2.17	0.69	0.10	0.004

Net income per month (1-5^a^)	733	3.86	1.10	0.05	0.151

Date of enrolment (0 = 1 Jan 1999, 7 = 1 Jan 2006)	913	2.35	1.82	0.03	0.341

AM medications in pre-study year (patient-months)	913	7.00	11.56	-0.03	0.335

Non-AM medications in pre-study year (patient-months)	913	10.91	14.89	-0.08	0.011

Psychotherapy in the pre-study year (sessions)	906	4.18	13.18	-0.01	0.809

Physiotherapy in the pre-study year (sessions)	913	8.73	17.96	-0.09	0.008

Disease duration (years)	913	7.84	9.23	-0.11	0.001

Disease severity at baseline (0-10)	902	6.68	1.74	0.10	0.004

Symptom Score at baseline (0-10)	913	6.01	1.77	0.47	< 0.001

Comorbid disorders (number)	913	1.76	1.34	-0.08	0.018

Chronic Disease Score (0-17 in this study) [65]	913	0.75	1.52	-0.08	0.014

SF-36 Physical Function Scale at baseline (0-100) [66]	909	75.46	23.35	0.04	0.240

SF-36 Role Physical Scale at baseline (0-100)	905	46.14	39.42	-0.06	0.071

SF-36 Role Emotional Scale at baseline (0-100)	900	50.19	41.64	-0.05	0.109

SF-36 Social Functioning Scale at baseline (0-100)	912	60.09	26.59	-0.09	0.008

SF-36 Bodily Pain Scale at baseline (0-100)	911	54.22	28.56	-0.05	0.129

SF-36 Vitality Scale at baseline (0-100)	910	38.55	18.81	-0.06	0.052

SF-36 Mental Health Scale at baseline (0-100)	910	54.21	19.37	-0.09	0.009

SF-36 General Health Scale at baseline (0-100)	902	50.65	19.34	0.00	0.917

SF-36 General Health Item at baseline (1 = excellent, 5 = poor)	908	3.58	0.77	-0.05	0.155

SF-36 Health Change Item at baseline^b^	910	3.25	1.07	0.12	< 0.001

SF-36 Physical Component Summary at baseline	887	43.22	10.52	0.01	0.876

SF-36 Mental Component Summary at baseline	887	38.20	12.28	-0.07	0.029

Center for Epidemiological Studies Depression Scale, German version at baseline (0-60) [67]	860	21.31	11.50	0.08	0.016

Physicians' clinical experience (years since qualification)	124^c^	18.20^c^	7.44^c^	-0.01	0.748

Number of patients enrolled by the physician	124^c^	7.36^c^	10.69^c^	0.06	0.073

Duration of consultation with physician at study enrolment (1-5^d^)	913	1.87	1.06	-0.01	0.826

Physician's therapy goal for the patient at study enrolment (1-6^e^)	911	2.78	1.23	0.07	0.042

Therapists' clinical experience (years since qualification)	123^e^	12.04^e^	^7.23e^	0.06	0.077

AM therapy in months 0-6 (therapy sessions)	905	8.79	8.71	0.06	0.062

AM medications in months 0-6 (patient-months)	911	6.57	7.83	-0.08	0.022

Non-AM medications in months 0-6 (patient-months)	911	6.33	8.22	-0.11	0.002

Psychotherapy in months 0-6 (therapy sessions)	906	2.51	7.67	-0.01	0.752

Physiotherapy in months 0-6 (therapy sessions)	909	3.91	9.74	-0.07	0.036

Correlation: Spearman-Rho correlation with dependent variable (Symptom Score difference 0-6 months).

^a^Income: documented as 1 = < 500€, 2 = 500€-900€, 3 = 900€-1250€, 4 = 1250€-1750€, 5 = > 1750€.

^b^SF-36 Health Change Item: 1 = much better now than one year ago, 5 = much worse now than one year ago.

^c^Numbers refer to physicians not patients.

^d^Duration of consultation: documented as 1 = 0-29 min, 2 = 30-44 min, 3 = 45-59 min, 4 = ≥60 min.

^d^6 = cured, 5 = symptom free without ongoing therapy, 4 = symptom free with ongoing therapy, 3 = symptom improvement, 2 = avoidance of progression, 1 = retardation of progression.

**Table 2:** Binomial independent variables

Variable	N	N	Correlation	
	
	Yes	Evaluable	R	P-value
Female gender	748	913	-0.04	0.175

Wage earner	32	913	-0.03	0.424

Living alone	167	909	0.04	0.220

Alcohol use daily	26	913	-0.02	0.535

Smoking daily	102	912	-0.01	0.715

Overweight (Body mass index > 25)	234	899	0.03	0.339

Permanent work disability pension or severe disability status	168	911	-0.03	0.300

Main diagnosis (International Classification of Diseases, 10^th ^Revision)				

• F00-F99 Mental/behavioural	278	913	0.02	0.584

• M00-M99 Musculoskeletal	184	913	-0.07	0.031

• G00-G99 Nervous system	69	913	0.03	0.365

• N00-N99 Genitourinary system	68	913	-0.04	0.267

• J00-J99 Respiratory system	66	913	0.08	0.019

• C00-D48 Neoplasms	56	913	0.05	0.104

• K00-K93 Digestive system	38	913	-0.01	0.691

• L00-L99 Skin and subcutaneous tissue	30	913	-0.03	0.419

• I00-I99 Circulatory system	29	913	-0.02	0.581

• E00-E90 Endocrine, nutritional, metabolic	25	913	-0.02	0.537

• Other diagnoses	70	913	0.01	0.732

Enrolment by primary care physician	757	913	-0.02	0.525

Previous treatment by physician	627	904	-0.03	0.391

Main AM therapy modality				

• Eurythmy therapy	447	913	-0.04	0.200

• Art therapy	190	913	0.02	0.571

• Rhythmical massage therapy	90	913	0.02	0.549

• Medical therapy	186	913	0.02	0.564

Therapy costs reimbursed	554	913	-0.03	0.377

Correlation: Spearman-Rho correlation with dependent variable (Symptom Score difference 0-6 months). All variables were coded as 0: no, 1: yes.

### Data collection

All data were documented with questionnaires. Questionnaires used at study enrolment were handed out by the physicians; follow-up questionnaires were administered from the study office by post. All questionnaires were returned in sealed envelopes to the study office. The physicians documented eligibility criteria, diagnosis, disease duration and severity, comorbid disorders, and therapy goals; all other items were documented by the patients. The patient responses were not made available to the physicians. The physicians were compensated 40 Euro per included and fully documented patient, while the patients received no compensation.

The data were entered twice by two different persons into Microsoft^® ^Access 97 (Microsoft Corp., Redmond, WA, USA). The two datasets were compared and discrepancies resolved by checking with the original data.

### Quality assurance, adherence to regulations

The study was approved by the Ethics Committee of the Faculty of Medicine Charité, Humboldt University, Berlin, Germany, and was conducted according to the Declaration of Helsinki and largely following the ICH Guideline for Good Clinical Practice E6. Written informed consent was obtained from all patients before enrolment.

### Data analysis

The data analysis was performed on all patients fulfilling the eligibility criteria, using PASW^® ^Statistics 18.0 (SPSS Inc., Chicago, Ill, USA) and StatXact^® ^5.0.3 (Cytel Software Corporation, Cambridge, MA, USA).

Diagnoses were coded according to the International Classification of Diseases, 10^th ^Revision. Medication use was assessed as the number of patient-months of use. For each medication, the number of patient-months was calculated as 'duration of use' × 'frequency of use' (F), where F = 1 for medication taken daily, 3-6 days per week or 1-2 days per week; F = 1/15 for medication taken 1-3 days per month; F = 0 for medication taken < 1 day per month. AM medications were defined as any medication produced by Abnoba Arzneimittel GmbH, Pforzheim, Germany; Helixor Heilmittel GmbH & Co, Rosenfeld, Germany; WALA Heilmittel GmbH, Eckwälden, Germany; or Weleda AG, Schwäbisch-Gmünd, Germany. Non-AM medications were defined as all other medications. The number of patient-months for all AM medications and all non-AM medications, respectively, was calculated as the sum of all patient-months in question.

Bivariate analyses were performed using Fisher's exact test for independent binominal data, t-test for continuous data, and Wilcoxon test for paired rank-ordered data; median differences with 95% confidence intervals (95%-CI) were estimated according to Hodges and Lehmann [42]. Bivariate correlations were calculated with Spearman-Rho. All tests were two-tailed. Significance criterion was p < 0.05. Since this was a descriptive study, no adjustment for multiple bivariate comparisons was performed [43].

For regression analysis, missing values were replaced by the group mean value (except in a sensitivity analysis, see below). Multinomial data were coded as dummy variables. A total of 61 independent variables were analysed (Tables 1, 2). The possibility of clustered patient sampling on the physician level was investigated by calculating intraclass correlation coefficients (ICC type 1,1 according to Shrout and Fleiss [44]) between the dependent variable (Symptom Score 0-6 month difference) and physicians. There was no evidence of clustering: (ICC = -0.51, p = 1.000). Likewise, there was no evidence of clustering on the therapist level (ICC = -0.52, p = 1.000). A multilevel analysis (physician and therapist level in addition to patient level) was therefore not considered necessary. Stepwise multiple regression analysis was performed with estimation of variance components by ordinary least squares. Criterion for inclusion of variables in the model was p < 0.05 and for exclusion p ≥ 0.10. Model assumptions were checked and verified [45,46]. For all variables included in the model the variance inflation factors were < 1.5, suggesting that multicollinearity was not a problem. Linearity, normality, and homogeneity of variances were checked with graphical methods. Cook's distance was used to identify influential observations, but no observations with significant influence were identified.

Five sensitivity analyses (SA) were performed to explore whether altered preconditions lead to relevant changes in the regression model:

• In SA1 missing values were not replaced, as in the main analysis (n = 913); instead the sample was restricted to patients with evaluable data for all 61 independent variables (n = 630).

• SA2 and SA3 were performed to validate the model. While the main analysis comprised all eligible patients, in SA2 this analysis was repeated on a random subsample of 75% of the original sample.

• SA3 was performed on the remaining 25% of the original sample. The variables retained in the final model of SA2 (a stepwise analysis), were forcibly entered blockwise into a regression model.

• In SA4 and SA5 the dependent variable differed from the main analysis and SA1-SA3 (the 0-6 month improvement of Symptom Score, n = 913): In SA4 the dependent variable was instead the 0-12 month improvement of Symptom Score (n = 840 evaluable patients).

• In SA5 the dependent variable was the 0-6 month improvement of the first ranked symptom of the 1-6 symptoms documented at baseline (instead of the average of all symptoms per patients, i. e. Symptom Score) (n = 906 evaluable patients). For this analysis, the baseline score of the first ranked symptom was substituted for Symptom Score as independent variable.

A few outliers with studentised residuals ≥ 3 standard deviations from zero were identified (main analysis, SA4 and SA5: each n = 1 outlier; SA1 and SA2: each n = 2 outliers). Outliers were retained in the main analysis and in SA 5 for the following reasons: in the main analysis the exclusion of the outlier affected results only minimally; in SA5 the exclusion of the outlier lead to the introduction into the model of a predictor variable (SF-36 role physical) which was not included in any other model. Outliers were excluded in SA1, SA2 and SA4 because in each case this exclusion lead to the disappearance from the model of a predictor variable not included in any other model (SA4: living alone) or not included in any other model but SA1 and SA2 (SA1 and SA2: enrolment by primary care physician).

## Results

### Patient enrolment

From 1 January 1999 to 31 December 2005, a total of 1,176 patients aged 17-75 years were assessed for eligibility. Of these patients, 913 fulfilled all eligibility criteria and were included in the analysis. Of the 263 patients who were not included, 156 did not have Symptom Score evaluable at 0 and 6 months and 107 patients were not included in the AMOS study for the following reasons: patients' baseline questionnaire missing (n = 42), patients' and physicians' baseline questionnaire dated > 30 days apart (n = 36), previous or ongoing use of AM therapy (n = 15), no informed consent (n = 9), other reasons (n = 5). Included (n = 913) and not included patients (n = 263) did not differ significantly regarding age, gender, diagnosis, disease duration, baseline disease severity or baseline Symptom Score.

A total of 124 physicians enrolled patients. These physicians did not differ significantly from eligible physicians without study patients (n = 215, excluding paediatricians) regarding the number of years in practice (mean ± standard deviation 18.2 ± 7.4 years vs. 20.1 ± 9.7 years, p = 0.087) and the proportion of primary care physicians (84.7% vs. 82.3%, p = 1.000). Significant differences were found regarding age (46.9 ± 7.1 years vs. 49.0 ± 8.3 years, p = 0.027, mean difference 2.1 years, 95% confidence interval [95%-CI] 0.2-3.9 years) and the proportion of male physicians (53.2% vs. 64.7%, p = 0.0497, odds ratio for male gender 1.61, 95%-CI 1.03-2.52).

The patients referred to AM art, eurythmy or rhythmical massage therapy were treated by 203 different therapists. Comparing these therapists to eligible therapists without study patients (n = 972), no significant differences were found regarding age (mean 48.9 ± 7.8 vs. 50.5 ± 9.8 years, p = 0.078), gender (83.3% vs. 80.7% women, p = 0.431) or the number of years since therapy school graduation (12.0 ± 7.2 vs. 13.3 ± 9.0 years, p = 0.144).

Each physician enrolled 1-4 patients (59.7%, n = 74/124 physicians), 5-9 patients (19.4%, n = 24) or ≥ 10 patients (21.0%, n = 26), with a median of 3.0 patients enrolled per physician (range 1-55 patients, interquartile range 2.0-8.0 patients, average 7.4 patients). For the whole AMOS sample enrolled in the same period (n = 1,568 children and adults) the corresponding number was average 10.2 patients enrolled per physician.

### Correlates of patient selection

For patients referred to new AM treatment with eurythmy, art or rhythmical massage therapy (see Methods, inclusion criterion 2B, n = 727 of 913 patients), it was investigated if the degree of patient selection by the physicians correlated with the physicians' therapy goal for the patient at study enrolment or with the Symptom Score 0-6 month difference. The degree of patient selection by the physicians was estimated for the whole AMOS study as follows:

• The patient recruitment into AMOS lasted from 1 July 1998 to 31 December 2005 (a total of 2,741 days). For each physician enrolling patients at least once in this period (n = 155 physicians), the physician's recruitment period was calculated as the number of days between the first and last enrolled patient. For physicians enrolling patients on more than one day (n = 130 physicians) the average recruitment period was 817 days, corresponding to 30.0% of the total recruitment period of 2741 days.

• In a survey of AMOS physicians conducted in November 2001, the physicians retrospectively documented the number of patients they had referred to new AM treatment in the past 12 months (corresponding to inclusion criterion 2B of the present analysis).

• For each physician with available data from this survey and enrolling patients on more than one day (n = 66 physicians), the degree of selection was calculated as the ratio (number of patients enrolled into AMOS and referred to new AM treatment in the physician's recruitment period)/(number of patients referred to new AM treatment according to the survey). The ratio was adjusted to a 12-month period. For the whole AMOS study it was thus estimated that the physicians enrolled 30.7% of the patients they referred to new AM treatment.

Of the 124 physicians enrolling patients into the present sample, 109 physicians enrolled patients referred to new AM treatment (n = 727 patients). Of these 109 physicians, 102 physicians enrolled patients on more than one day (with n = 720 patients referred to AM treatment), and 56 physicians (with n = 500 patients referred to AM treatment) also had available data on the degree of patient selection. For these 500 patients, the degree of patient selection of their physician was analysed for correlations: The degree of patient selection was significantly correlated with the physicians' therapy goal at baseline (Spearman-Rho = 0.12; p = 0.008) but not with the Symptom Score 0-6 month difference (Spearman-Rho = 0.01; p = 0.744).

### Patient description

The patients were recruited from 15 of 16 German federal states. Age groups were 17-29 years (9.2%, 84 of 913 patients), 30-39 years (26.9%), 40-49 years (34.8%), 50-59 years (15.3%), and 60-75 years (13.7%) with a mean age of 44.4 ± 12.1 years. A total of 81.9% (748/913) of the patients were women.

Most frequent main diagnoses were mental disorders (30.4%, 278 of 913 patients) and musculoskeletal diseases (20.1%) (Table 2). Further data on demographics, morbidity, and therapies are presented in Tables 1 and 2. Of the 1-6 symptoms documented per patient (see Methods), the first ranked symptom (mean 6.37 points at baseline) had a higher intensity than the remaining five symptoms (range of means 5.43 to 5.96 points, average of means 5.72 points) (p < 0.001). Likewise the 0-6 month improvement of the first ranked symptom (average 2.84 points, 95%-CI 2.67-3.02 points) was more pronounced than the improvement of the remaining symptoms (2.17 points, 95%-CI 1.99-2.34 points) (p < 0.001).

Symptom Score averaged 6.01 ± 1.77 points at baseline and improved by average 2.53 points (95%-CI 2.39-2.68 points, p < 0.001, n = 913) from 0 to 6 months, by 2.49 points (95%-CI 2.32-2.65 points, p < 0.001, n = 840 evaluable patients) from 0 to 12 months, and by 2.73 points (95%-CI 2.55-2.91 points, p < 0.001, n = 793) from 0 to 24 months.

At 6-month follow-up the physicians documented the highest therapy goal attained (rank ordered scale: see Methods section, with the additional response category "no goal attained"). Compared to the goal formulated by the physician at enrolment, a higher goal was attained in 13.6% of patients (n = 104 of 761 patients with physician follow-up data available), the same goal was attained in 44.7% (n = 340), and a lower goal or no goal was attained in 41.7% (n = 317); the Hodges-Lehmann estimate of median difference indicated that a 1.00 point lower goal was attained at follow-up compared to the goal aimed for at baseline (95%-CI 1.00-1.50 points; Wilcoxon signed-rank test: p < 0.001).

### Predictors of 0-6 month Symptom Score improvement

#### Variable not included in main analysis

One item of potential interest, 'duration of physician-patient-relationship' (i. e. how many years has the patient been treated by the study physician) was not included in the main analysis, since this item was only documented in a subset of patients enrolled after 15 May 2002 (n = 175). In these patients the duration of the physician-patient-relationship showed only a weak and non-significant correlation with the 0-6 month Symptom Score improvement (Spearman-Rho = -0.05, p = 0.524).

#### Main analysis

Of 61 analysed independent variables, 19 variables showed significant univariate correlations (p < 0.05) with the 0-6 month Symptom Score improvement, but only four of these variables had correlations with Spearman-Rho > ± 0.10 (baseline Symptom Score: r = 0.47; baseline SF-36 Health Change item: r = 0.12; disease duration: r = -0.11; use of non-AM medications in months 0-6: r = -0.11) (Tables 1, 2).

Stepwise multiple linear regression analysis yielded a significant model (F = 49,397; p < 0.001) which accounted for 32% of the variance (Tables 3) and included nine predictor variables. The strongest predictor was baseline Symptom Score, which alone accounted for 25% of the variance. The regression coefficients of the model (Tables 3, 4) can be clinically interpreted as follows (each interpretation presupposes that all other variables in the model are kept constant):

**Table 3:** Predictors of 0-6 month Symptom Score improvement: results of stepwise multiple regression analysis

Variable	R	**Adjusted R**^**2**^	B	Standard error of B	Beta	t-value	P-value
Intercept			-1.87	0.69		-2.73	0.007

1. Symptom Score at baseline (1-10)	0.50	0.25	0.74	0.04	0.58	20.16	< 0.001

2. SF-36 Physical Function Scale at baseline (0-100)	0.53	0.28	0.01	0.00	0.11	3.36	0.001

3. Education level (1 = low, 2 = intermediate, 3 = high)	0.54	0.29	0.31	0.09	0.10	3.46	0.001

4. SF-36 General Health Item at baseline (1-5)	0.55	0.30	-0.29	0.09	-0.10	-3.17	0.002

5. Main diagnosis J00-J99 Respiratory system (0: no, 1: yes)	0.56	0.31	0.77	0.24	0.09	3.14	0.002

6. Disease duration (years)	0.56	0.31	-0.02	0.01	-0.08	-2.70	0.007

7. Main diagnosis N00-N99 Genitourinary system (0: no, 1: yes)	0.57	0.32	-0.63	0.24	-0.07	-2.65	0.008

8. Physiotherapy in pre-study year (sessions)	0.57	0.32	-0.01	0.00	-0.07	-2.54	0.011

9. Physician's therapy goal at baseline (6 = cured, 1 = retardation of progression)	0.57	0.32	0.12	0.05	0.06	2.26	0.024

R: Multiple correlation coefficient. Adjusted R^2^: Adjusted coefficient of multiple determination. B: Regression coefficient. Beta: Standardised regression coefficient.

1. Symptom Score at baseline: For each 1.00 point increase in baseline Symptom Score (increase means worse symptoms) the 0-6 month Symptom Score improvement will increase by average 0.74 points (95%-CI 0.66-0.81).

2. SF-36 Physical Function at baseline: For each 1 point increase in the baseline SF-36 Physical Function Scale (increase means better physical function) the improvement will increase by average 0.01 points. E. g. in patients with an increase of 20 points (approximately one-half standard deviation of this scale) the Symptom Score improvement will increase by 0.20 points.

3. Education level: Patients with high education level will have average 0.31 points (95%-CI 0.14-0.49) larger improvement than patients with intermediate education level, and these will have 0.31 points larger improvement than patients with low education level.

4. SF-36 General Health at baseline: For each 1 point decrease in the baseline SF-36 General Health item (decrease means better general health) the improvement will increase by average 0.29 points (95%-CI 0.11-0.47).

5. Main diagnosis J00-J99 Respiratory System: Patients with this diagnosis will have average 0.77 points (95%-CI 0.29-1.25) more improvement than all other patients.

6. Disease duration: For each year of disease duration prior to study enrolment, the improvement will decrease by 0.02 points (95%-CI 0.01-0.03).

7. Main diagnosis N00-N99 Genitourinary System: Patients with this diagnosis will have average 0.63 points (95%-CI 0.16-1.10) less improvement than all other patients.

8. Physiotherapy in the pre-study year: For each physiotherapy session in the pre-study year the improvement will decrease by average 0.01 points (95%-CI 0.00-0.02). E. g. patients with 12 physiotherapy sessions will have 0.12 points less improvement than patients without physiotherapy.

9. Physicians' therapy goal at baseline: For each higher step on the rank-ordered scale of therapy goals (see Methods for details) the improvement will increase by 0.12 points (95%-CI 0.02-0.22).

#### Sensitivity analyses

Five sensitivity analyses (SA) were performed (see Methods for details). All SA yielded significant models with 7-11 independent variables, together accounting for 31%-33% of the variance (Tables 4 and 5). Of the nine variables in the main model, three variables remained significant predictors in all five SA (baseline Symptom Score [in SA5: first ranked symptom at baseline], baseline SF-36 Physical Function, baseline SF-36 General Health), two variables remained significant predictors in four SA (education level, physician's therapy goal at baseline: SA1+2+4+5), and one variable remained a significant predictor in three SA (respiratory disorders: SA1+2+5). Seven of the nine variables in the main model (not the two diagnoses) also predicted Symptom Score improvement after 12 months (SA4), and eight variables (not disease duration at baseline) also predicted the improvement of the first ranked baseline symptom after 6 months (SA5).

**Table 4:** Sensitivity analyses (SA): Regression coefficients with 95% confidence intervals

Variable	Main analysis	SA1: Evaluable data for all variables	SA2: 75% subsample	SA3: Remaining 25%, variables from SA2	SA4: 0-12 month improvement	SA5: Dependent variable First Symptom
1. Symptom Score at baseline (0-10)*	0.74 (0.66 to 0.81)	0.75 (0.66 to 0.83)	0.73 (0.65 to 0.81)	0.74 (0.59 to 0.90)	0.75 (0.67 to 0.83)	0.72 (0.64 to 0.79)

2. SF-36 Physical Function Scale at baseline (0-100)	0.01 (0.004 to 0.02)	0.01 (0.01 to 0.02)	0.01 (0.003 to 0.02)	0.01 (0.002 to 0.03)	0.01 (0.002 to 0.02)	0.01 (0.0003 to 0.02)

3. Education level (1-3)	0.31 (0.14 to 0.49)	0.32 (0.10 to 0.54)	0.35 (0.15 to 0.56)	0.31 (-0.06 to 0.69)	0.30 (0.11 to 0.50)	0.44 (0.22 to 0.66)

4. SF-36 General Health Item at enrolment (1-5)	-0.29 (-0.47 to -0.11	-0.26 (-0.48 to -0.05)	-0.23 (-0.43 to -0.03)	-0.63 (-1.03 to -0.23)	-0.24 (-0.44 to -0.05)	-0.23 (-0.44 to -0.01)

5. Main diagnosis J00-J99 Respiratory system**	0.77 (0.29 to 1.25)	0.68 (0.13 to 1.23)	0.76 (0.24 to 1.27)	0.65 (-0.46 to 1.77)	---	0.67 (0.10 to 1.25)

6. Disease duration (years)	-0.02 (-0.03 to -0.01)	-0.02 (-0.03 to -0.0005)	---	---	-0.02 (-0.03 to -0.002)	---

7. Main diagnosis N00-N99 Genitourinary system**	-0.63 (-1.10 to -0.16)	-0.62 (-1.18 to -0.07)	---	---	---	-0.72 (-1.28 to -0.16)

8. Physiotherapy in pre-study year (sessions)	-0.01 (-0.02 to -0.002)	---	---	---	-0.01 (-0.02 to -0.01)	-0.01 (-0.02 to -0.004)

9. Physician's therapy goal at baseline (1-6)	0.12 (0.02 to 0.22)	0.18 (0.06 to 0.30)	0.17 (0.06 to 0.28)	0.01 (-0.22 to 0.23)	0.18 (0.07 to 0.29)	0.27 (0.15 to 0.40)

10. Psychotherapy in pre-study year (sessions)	---	-0.01 (-0.02 to -0.004)	---	---	---	---

11. Wage earner**	---	---	0.90 (0.18 to 1.62)	-0.66 (-2.28 to 0.97)	---	1.04 (0.24 to 1.84)

12. Non-AM medications in month 0-6 (patient-months)	---	---	---	---	-0.03 (-0.05 to -0.01)	-0.02 (-0.04 to -0.002)

13. Main diagnosis C00-D48 Neoplasms**	---	---	---	---	---	0.70 (0.08 to 1.32)

*SA5: First ranked symptom at enrolment. **: 0: no, 1: yes. ---: Variable not included in the model.

**Table 5:** Sensitivity analyses (SA): Summary of models

Analysis	N patients	Included variables	**Adjusted R**^**2**^ **1**^**st **^**variable**	**Adjusted R**^**2**^ Full model	ANOVA P-Value
Main analysis	913	1,2,3,4,5,6,7,8,9	0.25	0.32	< 0.001

SA1: Patients with evaluable data for all variables	630	1,2,9,3,10,4,5,7,6	0.24	0.33	< 0.001

SA2: 75% random subsample	698	1,2,5,3,9,11,4	0.27	0.33	< 0.001

SA3: Remaining 25%, variables from SA2 model	213	1+2+5+3+9+11+4	Not applicable	0.32	< 0.001

SA4: Dependent variable Symptom Score 0-12 months	839	1,2,12,8,9,3,4,6	0.23	0.33	< 0.001

SA5: Dependent variable First Symptom 0-6 months	906	1*,2,9,3,8,7,11,4,5,13,12	0.23*	0.31	< 0.001

Included variables: Variable numbers from Table 4. *: Variable 1 = First ranked symptom at enrolment. Adjusted R^2^: Adjusted coefficient of multiple determination. ANOVA p-value: Analysis of variance with significance test for total model.

Among the SA using the improvement of Symptom Score as dependent variable (SA1-4), all had baseline Symptom Score as first ranked variable in the model (not applicable for SA3, where all variables were included blockwise), this variable alone accounting for 23%-27% of the variance. The regression coefficient for baseline Symptom Score (main analysis: 0.74 point more improvement per 1.00 point increase in baseline Symptom Score) showed very little variation in SA1-4 (range 0.72-0.75 points). In SA5 the first ranked symptom had been substituted for Symptom Score as independent (baseline score) and dependent variable (0-6 month change); results were very similar to the other analyses, with the first symptom being first ranked variable in the model, accounting for 23% of the variance, and with a similar regression coefficient of 0.72.

All SA had baseline SF-36 Physical Function as second ranked variable (again not applicable for SA3) with a regression coefficient of 0.01 in all analyses.

The regression coefficients for the other predictor which was significant in all SA (baseline SF-36 General Health) also varied relatively little in SA1+2 and SA4+5 but showed more variation in SA3 (Table 4).

In all SA one variable (SA1-4) or three independent variables (SA5) which had not been included in the main model were now included, altogether four new independent variables (Table 4):

• In SA1 psychotherapy in the pre-study year was a negative predictor.

• In SA2 and SA5 the variable 'wage earner' was a positive predictor. However, in SA3 this prediction was reversed and no longer significant.

• In SA4 and SA5 the use of non-AM medications in months 0-6 was a negative predictor.

• In SA5 a main diagnosis of neoplasms was a positive predictor.

• If outliers were retained in SA1 and SA 2 (see Methods for details) enrolment by primary care physician was a positive predictor in these two SA. Results (not shown elsewhere) indicate that patients enrolled in primary care will have 0.40 point more improvement than patients enrolled in specialist practice or outpatient clinics.

## Discussion

### Major findings

This is the first multivariate predictor analysis of long-term outcome following AM treatment for chronic non-cancer indications in adults. In 913 adult outpatients in Germany, improvement after 6 and 12 months was positively predicted by higher baseline symptom severity, better physical function, better general health, and (in most analyses) higher education level and a higher therapy goal at baseline. The remaining variables were not significant predictors or were not retained when repeating the analysis on random subsamples of the original sample. Baseline symptom severity was the strongest predictor, accounting for 25% of the variance (total model 32%).

### Strengths and limitations

Strengths of this analysis include a large sample size, validation of the main model in subsamples, and a high representativeness due to the participation of 22% of eligible AM physicians and therapists in Germany. The participating AM physicians and therapists resembled eligible but not participating physicians and therapists with respect to demographic characteristics, and the included patients resembled not included patients regarding baseline characteristics [20]. These features suggest that the study mirrors contemporary AM use in outpatient settings to a high degree. The analysis also comprised a detailed assessment of socio-demographics, disease status, co-morbidity, disability, depression, AM therapies, adjunctive therapies, and therapist factors. Other factors of potential interest, such as psychosomatic [22] or autonomic [47] self-regulation, patient expectations [48,49], and perceived quality of the physician-patient relationship and therapist-patient relationship could not be assessed, as they were not documented in the study.

Since the study had a long recruitment period, the study physicians were not able to screen and enrol all eligible patients (criteria: see Methods section). For patients referred to new AM therapies, it was estimated that physicians enrolled 31% of eligible patient into AMOS. This selection could bias results if physicians were able to predict therapy response and if they preferentially screened and enrolled such patients for whom they expected a particularly favourable outcome. The available data suggest that the physicians may to some degree have selected patients with an expected positive outcome: the degree of patient selection (= the proportion of eligible vs. enrolled patients) showed a significant albeit weak correlation (Spearman-Rho 0.12) with physicians' therapy goal at baseline. However, the physicians' ability to predict future outcomes was far from perfect: their therapy goals were not attained in 42% of patients, and therapy goals at baseline showed only a weak correlation with the Symptom Score improvement at 6-month follow-up (Spearman-Rho 0.07). Moreover, the degree of patient selection in itself was not correlated with the Symptom Score improvement. Thus, if any patient selection on account of expected therapy response did occur, the analysed data do not suggest that such a selection affected clinical outcomes. Notably, our analysis of patient selection has several limitations: it did not include patients starting AM medical therapy (inclusion criterion 2A, see Methods: 20% of the present sample); the analysis was based on a retrospective documentation of the number of eligible patients; and, because of non-response and other reasons, 31% of patients referred to new AM therapy had no available data on the degree of selection. We cannot, therefore, exclude an effect of patient selection on the predictions found in the present analysis.

The target of the present predictor analysis was pre-post improvement after 6 and 12 months in patients receiving AM therapy for chronic disease. Long-term improvement is a useful outcome measure in rehabilitation research, but pre-post improvement may of course have other causes apart from the therapy. Since the present analysis was based on data from a single-arm study of AM therapy, the impact of such causes could not be estimated through direct comparison to a control group.

The independent variable used in the main analysis of this paper, Symptom Score, is a compound score of the 1-6 most relevant symptoms in each patient, measured on 0-10 point numerical rating scales. Numerical rating scales are valid, reliable and responsive measures of pain [41], and 0-10 point numerical rating scales have been extensively used to measure other symptoms than pain [50-53] as well as global disease severity [54]. In addition, compound scores, based on several 0-10 point numerical rating scales, have been constructed to measure individualised quality of life [55]. The compound Symptom Score used in this study has not been validated. However, when Symptom Score was substituted with the single most important baseline symptom in each patient, measured on a single numerical rating scale (SA5), results were very similar to the main analysis.

Our regression models are of course, like all statistical models, imperfect representations of reality [56]; they do not prove causality, and residual confounding cannot be ruled out. A limitation of stepwise multiple linear regression analyses on a large dataset using a large number of independent variables is the possibility of chance findings from modelling random variations [57]. Another limitation of the present analysis is that it was not pre-specified in the original protocol. Altogether, this secondary predictor analysis of data from a pre-post study does not allow for causal conclusions; the results are hypothesis generating and need verification in subsequent studies.

### Interpretation, comparison to other studies

The strongest outcome predictor in this analysis was baseline symptoms. The more outspoken improvement among patients with worse baseline symptoms can have several causes, such as more room for improvement and more regression to the mean with higher score values, the hello-goodbye effect, and a higher patient motivation with therapies working better at higher symptom levels [39,58]. Physical function as well as general health at baseline predicted future improvement in the opposite direction than baseline symptoms (i. e. better physical function and better general health predicted more improvement while lower symptom intensity predicted less improvement). These findings are plausible, since AM aims to mobilise self-healing capacities [9,10] and since patients with better physical fitness and/or general health may have more such capacities than patients with poor fitness and health, independently of symptom severity. Good general health and fitness might thus enhance natural as well as therapy-induced recovery.

A higher education level was a significant positive predictor of improvement in the main analysis and in four out of five sensitivity analyses. Well educated patients may have more motivation or capacity to engage in artistic (eurythmy exercises, art therapy) and self-reflective therapy forms (AM consultations). However, the 150 patients with low education levels in this analysis also had a significant and clinically relevant improvement of average 2.05 points.

The physicians' therapy goal for the patients at baseline was also a significant positive outcome predictor in five out of six analyses. Therapy goals may reflect physicians' prognostic assessment of patients' future therapy response, as discussed above. In this respect, physicians' observation of patients may yield prognostic information in addition to the information provided by the other predictors in our analysis. In addition, higher therapy goals may reflect the physicians' healing intentions [8,9] and a general therapeutic optimism.

The patients in this analysis were treated for a range of diagnoses. Among the ten most common diagnosis groups the only clearly significant outcome difference was a more outspoken 0-6 month improvement in patients with respiratory disorders (n = 66 patients, thereof 36 patients with asthma). Notably, a respiratory disorder was no significant predictor of improvement in the 25% subsample (SA3) and after 12 months (SA4). On the other hand, the point estimate for the regression coefficient in SA3 (0.65 points more improvement in patients with respiratory disorder than in other patients) was similar to the estimates in the main analysis and SA1-2 (range 0.67-0.77). When comparing different diagnoses one should take into account that symptom improvement, although documented uniformly with Symptom Score in all patients, may still have different meanings in different diagnosis groups. Predictor analyses restricted to individual diagnosis groups might of course yield results differing from the present analysis.

Negative predictions were found for increasing disease duration, genitourinary disorders, and physiotherapy in the pre-study year. These predictions should be interpreted with considerable reserve, since each of them was only reproduced in two out of five sensitivity analyses. The negative prediction by physiotherapy was contrary to our a-priori assumptions and may suggest that physiotherapy is an independent marker of chronicity and therapy refractoriness, rather than being harmful in itself. Other variables (psychotherapy in pre-study year, non-AM medication use in month 0-6, wage earner, neoplasm as main diagnosis) were only significant predictors in 1-2 sensitivity analysis and not in the main analysis and should therefore be interpreted with extreme caution.

Apart from the AMOS study, two other studies have investigated associations between patient characteristics and outcome of comprehensive AM treatment for chronic disease in adults [24,59]: In a study of inpatient AM treatment for patients with advanced epithelial cancer, improvements in quality of life were found among patients with good and bad performance status, respectively, but the two groups showed improvements in different quality of life domains [24]. In a Swiss study of adult primary care patients starting AM treatment for various disorders (thereof 53% with disease duration > 3 months), patient satisfaction and perceived benefit at four-week follow-up were independent of age and gender [59]. The latter finding was confirmed in our analysis of a German predominantly primary care sample. The predictions of improvement by higher baseline symptoms and shorter disease duration in this analysis of adult AMOS patients were also found in a corresponding predictor analysis of children in the AMOS study [27]. These two multivariate analysis also confirm previous univariate analyses from the AMOS study, showing similar improvement among different AM therapy modality groups [20,34-37]. This finding may be due to a predominant effect common to all AM therapy modalities. The opposite is also possible; different therapy modalities may have specific effects and still work comparably well for the respective patient groups.

Some of the significant outcome predictors in this analysis of AM treatment have also been identified as significant predictors of outcome following other complementary therapies for chronic conditions: baseline symptom intensity [48,60-63], disease duration [62], education [60,61], and baseline SF-36 scores [48,60,61]. However, education was not a significant outcome predictor in other studies of complementary therapies [49,64].

Regarding the SF-36 Health Survey, we analysed all eight SF-36 scales, the two SF-36 summary scores, and two further SF-36 items as independent variables, whereby the Physical Function scale and the General Health item were significant predictors. Several outcome prediction analyses of other complementary therapies have included one or both SF-36 summary scores but none of the remaining SF-36 scales or items as independent variables [48,60,61]. In these analyses, baseline SF-36 Physical Component Summary score [48,60,61] and baseline SF-36 Mental component summary score [60,61] were significant positive predictors of intermediate outcomes (discharge from inpatient rehabilitation [48] and three-month follow up [60,61], respectively). When the present analysis was repeated, retaining the two SF-36 summary scores but excluding the remaining SF-36 scales and SF-36 items from the independent variables, both the SF-36 Physical and Mental Component Summary scores emerged as significant positive predictors for the 0-6 month Symptom Score improvement.

Some studies of other complementary therapies have identified further outcome predictors which were not significant predictors in the present study: age [60,61,64], gender [60,61], and the number of therapy sessions [48]. However, in other studies of complementary therapies, age [49] and gender [49,64] were not significant predictors, like in the present study.

## Conclusions

This is the first multivariate predictor analysis of long-term outcome following AM treatment for chronic non-cancer indications in adult outpatients. Symptom improvement after 6 and 12 months was positively predicted by higher baseline symptom severity, better physical function, better general health, and in most analyses a higher education level and a higher therapy goal at baseline. A number of other variables were not associated with the outcome. This secondary predictor analysis of data from a pre-post study does not allow for causal conclusions; the results are hypothesis generating and need verification in subsequent studies.

## List of abbreviations

±: standard deviation; 95%-CI: 95% confidence interval; AM: Anthroposophic medicine; AMOS: Anthroposophic Medicine Outcomes Study; SA: Sensitivity analysis.

## Competing interests

Within the last five years HJH and GSK have received restricted research grants from the pharmaceutical companies Wala and Weleda, who produce AM medications. Otherwise all authors declare that they have no competing interests.

## Authors' contributions

HJH, CMW, GSK, SNW, and HK contributed to study design. HJH, AG, and HK contributed to data collection. HJH, and HK wrote the analysis plan, HJH and AG analysed data. HJH was principal author of the paper, had full access to all data, and is guarantor. All authors contributed to manuscript drafting and revision and approved the final manuscript.
